# Supplementary material for: Small-scale farming in drylands: New models for resilient practices of millet and sorghum cultivation
Source: PLoS One. 2023 Feb 2;18(2):e0268120. doi: 10.1371/journal.pone.0268120 (PMC9894398; doi:10.1371/journal.pone.0268120)
Supplement: S1 Appendix — (PDF) [file pone.0268120.s001.pdf]

Small-scale farming in drylands: new models for resilient practices of millet and sorghum cultivation Supplementary Material

immediate

ABSTRACT

This document contains supplementary information (extended results), figures and tables.

Extended results

Entries in the ethnographic database could be classified in 6 groups according to their crop package: communities that exclusively cultivate finger millet (16.7%), pearl millet (6.9%) or sorghum (30.6%); communities that cultivate both finger millet and sorghum (15.3%); communities that cultivate both pearl millet and sorghum (20.8%); communities that cultivate all 3 crops (9.7%). Noteworthy, no community showed exclusive cultivation of the two millets alone. Finger millet cultivation practices were identified by cross-tabulation as Extensive-Rainfed (56.6%), Intensive-Rainfed (36.7%) and Intensive-Irrigated agriculture (6.7%). The results of cross tabulations for pearl millet cultivation were similar to that of finger millet, but with a higher presence of irrigated systems: 55.6% entries were classified as Extensive-Rainfed, 29.6% as Intensive-Rainfed and 14.8% as Intensive-Irrigated agriculture. No cases of casual agriculture or décrue watering were identified amongst either finger or pearl millet producers. Finally, sorghum agriculture featured a higher rate of diversity: along with Extensive-Rainfed (40%), Intensive-Rainfed (36.4%) and Intensive-Irrigated (10.9%), 2 additional groups were identified by cross tabulation as Casual-Rainfed (7.3%) and Intensive-Décrue (5.4%), for a total of 5 combinations for sorghum cultivation. In no instance, casual agriculture was observed to be combined with décrue or irrigated watering regimes, neither was extensive agriculture.

Modeling Variability

Global RDAs showed rather high explanatory potential in all cases: adjusted-R^2 values scored 56.3% for crop selection, 64.4% for finger millet, 100% for pearl millet and 71.8% for sorghum cultivation respectively. However, the models were only statistically significant for crop selection and sorghum cultivation datasets -the statistical significance of pearl millet was not tested as there was no residual fraction. The examination of variance inflation factors showed very high collinearity amongst the predictors included in the global RDAs. As a result of forward selection, the most significant predictors amongst explanatory datasets were identified for each model:

- Six variables appeared as the most relevant for crop selection (p-values < 0.05): mean top-soil volumetric water content at 15 kPa, mean top-soil pH, variance of mean temperature of the warmest quarter, mean global horizontal irradiance, variance of subsoil clay content and mean precipitation seasonality.
- The most significant variables for finger millet cultivation were: mean subsoil sulphur content, mean precipitation concentration index and top-soil mean phosphorus content.
- The most significant variables for pearl millet cultivation were: variance of temperature seasonality, variance of top-soil volumetric water content at 33 kPa, mean subsoil gravel content, mean top-soil clay content, mean duration of the growing cycle, the mean temperature of wettest quarter, variance of top-soil organic carbon content, variance of top-soil silt content and mean temperature during the driest quarter.
- The most significant variables for sorghum cultivation were: the mean of growing cycle duration, the variance of both top-soil and subsoil cation exchange capacity and the mean soil organic carbon.

Reduced models using forward selection predictors explained almost as much adjusted proportion of the total variance as global RDAs for crop selection (54.9%) and finger millet cultivation (60.7%). However, the constrained variance of pearl millet and sorghum cultivation was reduced to 87.8% and 24% respectively. Still, a clear improvement in statistical significance was achieved, as all reduced models passed the 1000 permutation test. Furthermore, all variables were found to be statistically significant and VIF analysis showed them to be independent to one another in all reduced models. Models were drawn using

triplots representing the 2 first RDA axes (Figure 4). These were always statistically significant except for sorghum cultivation, in which the second axis did not pass the permutation test and only the variability retained by the first axis was considered during the analysis even though we show both in Figure 4d. Model coefficients and ordination scores results showed the presence of finger millet cultivation to be mainly related to areas with higher values of mean top-soil volumetric water content at low suction pressure and variance of mean temperature of the warmest quarter (Figure 4a). Extensive finger millet cultivation was found to be highly related to precipitation concentration index, whereas intensive systems appeared linked to the content of top-soil phosphorus (Figure 4b). The use of irrigation in intensive regimes was shown to be mainly associated with subsoil sulphur content. Next, the choice of pearl millet appeared related to greater irradiance and precipitation seasonality, but also to variance in subsoil clay content in a lesser degree (Figure 4a). Extensive-rainfed systems were associated with longer growing cycles, as well as with higher values of top-soil clay content and variance of top-soil organic carbon content. Intensive agriculture of pearl millet was carried out by communities in regions which scored high in subsoil gravel content, mean temperature during the driest quarter and variance of top-soil water content at mid suction pressure. Rainfed regimes amongst intensive systems also depended on the two latter variables, whereas greater values of variance of temperature seasonality, mean temperature of wettest quarter and variance of top-soil silt content appeared in relation with irrigated systems (Figure 4c). Lastly, sorghum cultivation showed its higher limitation to be top-soil mean pH (Figure 4a). As for pearl millet, all rainfed sorghum regimes showed association with extended growing cycles. Casual-rainfed sorghum agriculture was identified in areas featuring elevated variance of cation exchange capacity (both topsoil and subsoil), whereas extensive-rainfed was linked to regions where only the latter was considerable. All intensive systems, regardless of watering regimes, appeared associated with communities inhabiting regions high in top-soil cation exchange capacity but also with higher soil organic carbon content per hectare, the main difference being décrue and irrigated agriculture not showing direct association with the duration of the growing cycles (Figure 4d).

Spatial Analysis and Variation Partitioning

Linear trend analysis by RDA revealed statistically significant models for crop selection and pearl millet cultivation variables, accounting for 15.4% and 25.8% of total variance respectively. XY coordinates were not included in the variation partitioning analysis of finger millet and sorghum cultivation as they did not pass the permutation test. None of the RDAs performed with dbMEMs were found to be statistically significant, nor was any dbMEM selected by means of FS, hence indicating a total absence of spatial autocorrelation in both finger millet and sorghum cultivation datasets. As a result, dbMEMs were not included in VP analysis. For crop selection, variation partitioning results showed significant effects of physio-climatic (PC), edaphic (ED) and spatial (XY) components on the variability of the study agricultural package (19.3%, 39.5% and 17.8% of the total inertia). When looking at their unique effects, the explained variance declined to 15.6%, 24.1% and 0.4% respectively. More importantly, the XY pure fraction failed to pass the test for statistical significance, hence pointing to the absence of spatial patterns in the crop selection dataset. By contrast, both physio-climatic and edaphic pure fractions significantly explained a combined 39.7% of the total inertia, and their shared fraction was almost non-existent. It is worth to note that a 12% of the variance retained by edaphic factors was also explained by the spatial component (Figure 2a, main text), thereby pointing to the existence of a linear trend amongst edaphic variables. Also, 4% of the total inertia was shown to be shared by all physio-climatic, edaphic and spatial predictors.

Next, VP analysis identified the impact of both physio-climatic and edaphic components on finger millet cultivation data to be statistically significant: the former retaining 22.9% of the total variability whereas the latter explained 46.1%. No shared fraction was identified between them (Figure 2b, main text). In the case of pearl millet cultivation, a component related to the duration of the plant growing cycle (GC) was also detected along with the physio-climatic, edaphic and spatial components. Growing cycles explained 7.9% of the total inertia, and was found to be barely relevant statistically ($p = 0.047$). As for the other components, they retained 32.8%, 27.3% and 25.8% of the variance and all were found to be statistically significant. On the one hand, the unique contribution of the growing cycle, physio-climatic and edaphic components was proved to be statistically meaningful and they accounted for 13.3%, 22.8% and 52.3% respectively. On the other hand, the pure effect of the spatial component was proven to hold no fraction of the total inertia, adding no constrained variance to the model and thereby showing the absence of spatial autocorrelation amongst the pearl millet cultivation dataset. Nonetheless, it featured important shared fractions with the rest of the components (Figure 2c, main text), showing the existence of linear spatial trends amongst them, especially in the physio-climatic fraction (35.1%). Finally, both growing cycle and edaphic components were found to significantly explain 8.4% and 16% of the total variability in sorghum cultivation, of which 0.4% was found to be shared by both fractions (Figure 2d, main text).

Model Validation using Ethnographic Observations

All four models were found to be capable of predicting their own training response datasets, and hence can be considered as valid models (Figure 5). The crop selection model showed 86.6% accuracy and a F1-score of 0.869, with precision and recall featuring values of 0.88 and 0.857 respectively. 95% accuracy was obtained for the finger millet cultivation model, whereas the

prediction of the pearl millet training data was 100% accurate. All their performance measures scored 0.95 and 1 respectively. Finally, the modeling of sorghum cultivation practices showed 78.2% accuracy and a F1-score of 0.723. In this case, recall was found to be larger (0.855) than precision (0.627) indicating a higher rate of false positives amongst the predictions.

The capacity of the models to correctly predict real data was tested at both individual and cultural levels with the data collected during ethnography. The modes of each predictor were used in order to create response datasets of the cultivation of all 3 study cereals for the Tigrinya, Sindhis and Sudan Arab as cultures. Descriptive statistics of the testing datasets are presented in Table 2 in the main text.

All reduced models scored between 60% and 80% accuracy when predicting individual cases (Figure 6a). Interestingly, the models F1-score (Figure 6b) remained similar to accuracy for crop selection, as well as for finger millet and pearl millet cultivation models. However, the sorghum cultivation model classification strength (F1-score) was lower than its accuracy by 8% due to a higher rate of false positives (0.4) than false negatives (0.233). Regarding the prediction of the testing cases as cultures (individuals mode), the reduced models showed an accuracy of 77.8% for crop selection, 100% for finger millet, 50% for pearl millet and 83.3% for sorghum (Figure 6c). Again, F1-scores (Figure 6d) for crop selection, finger millet and pearl millet cultivation models featured almost no change with respect to accuracy, whereas the sorghum cultivation model also showed lower precision (0.714) than recall (0.833).

Supplementary Tables

Table 1. Table S1. Variables considered (definitions and references)

Variable	Definition	References
FM Cultivation	Presence of finger millet (Eleusine coracana Gaertn.) production	eHRAF
PM Cultivation	Presence of pearl millet (Pennisetum glaucum (L.)R.Br.) production	eHRAF
SB Cultivation	Presence of sorghum (Sorghum bicolor (L.) Moench) production	eHRAF
Casual Agriculture	Slight or sporadic cultivation of food or other plants incidental to a primary dependence upon other subsistence practice	[1]
Extensive Agriculture	Or shifting cultivation, as where new fields are cleared annually, cultivated for a year or two, and then allowed to revert to forest or brush for a long fallow period	[1]
Intensive Agriculture	On permanent fields, utilizing fertilization by compost or animal manure, crop rotation, or other techniques so that fallowing is either unnecessary or is confined to relatively short periods	[1]
Rain-fed Agriculture	Water is provided by rainfall alone (directly or as run-off), cultivation occurs far from any permanent water sources and without any water harvesting	[2]
Décrue Agriculture	Water is provided by natural inundation, typically from major river systems (floodplain cultivation)	[2]
Irrigated Agriculture	Water is provided to crops at regular intervals throughout the growing season by human intervention	[2]
Duration of FM/PM/SB growing cycle	Mean growing cycle duration and variance	eHRAF

Table 2. Table S2. Summary of the environmental variables used in the study. Sources: Global Multi-resolution Terrain Elevation Data[3]; Global Solar Atlas [4, GSA]. Global Aridity and PET database[5]; WorldClim[6]; Global Soil Organic Carbon Map[7]; Global Soil Dataset for Earth System Models[8]

Environmental variables	Abbreviation
Altitude	ALT
Slope	SLO
Insolation time	INS
Global Horizontal Irradiance	GHI
Aridity Index	AI
Precipitation Concentration Index	PCI
Annual Mean Temperature	BIO1
Mean Diurnal Range (Mean of monthly (max temp - min temp))	BIO2
Isothermality (BIO2/BIO7) (* 100)	BIO3
Temperature Seasonality (standard deviation *100)	BIO4
Max Temperature of Warmest Month	BIO5
Min Temperature of Coldest Month	BIO6
Temperature Annual Range (BIO5-BIO6)	BIO7
Mean Temperature of Wettest Quarter	BIO8
Mean Temperature of Driest Quarter	BIO9
Mean Temperature of Warmest Quarter	BIO10
Mean Temperature of Coldest Quarter	BIO11
Annual Precipitation	BIO12
Precipitation of Wettest Month	BIO13
Precipitation of Driest Month	BIO14
Precipitation Seasonality (Coefficient of Variation)	BIO15
Precipitation of Wettest Quarter	BIO16
Precipitation of Driest Quarter	BIO17
Precipitation of Warmest Quarter	BIO18
Precipitation of Coldest Quarter	BIO19
Soil Organic Carbon	SOC
Bulk density (Top-soil and subsoil)	BD1 and BD2
Clay content (Top-soil and subsoil)	CLAY1 and CLAY2
Silt content (Top-soil and subsoil)	SILT1 and SILT2
Sand content (Top-soil and subsoil)	SAND1 and SAND2
Gravel content (Top-soil and subsoil)	GRAV1 and GRAV2
Cation Exchange Capacity (Top-soil and subsoil)	CEC1 and CEC2
Electrical Conductivity (Top-soil and subsoil)	ECE1 and ECE2
pH (H2O) (Top-soil and subsoil)	PH1 and PH2
Organic Carbon (Top-soil and subsoil)	OC1 and OC2
Total Potassium (Top-soil and subsoil)	TK1 and TK2
Total Nitrogen (Top-soil and subsoil)	TN1 and TN2
Total Phosphorus (Top-soil and subsoil)	TP1 and TP2
Total Sulphur (Top-soil and subsoil)	TS1 and TS2
Volumetric water content at -10 kPa (Top-soil and subsoil)	WC11 and WC12
Volumetric water content at -33 kPa (Top-soil and subsoil)	WC21 and WC22
Volumetric water content at - 1500 kPa (Top-soil and subsoil)	WC31 and WC32

Table 3. Table S3 List of references by eHRAF community

ID	Culture	Region	References	Field dates
1	Amhara	Eastern Africa	9, 10	1953-1970
2	Azande	Central Africa	11–15	1900-1953
3	Bagisu	Eastern Africa	16	1965-1969
4	Bambara	Western Africa	17–20	1945-2000
5	Barundi	Central Africa	21, 22	1911-1957
6	Bemba	Southern Africa	23–25	1930-1934
7	Bena	Eastern Africa	26	1931-1933
8	Central Thai	Southeast Asia	27	1960-1970
9	Dogon	Western Africa	28–32	1931-1991
10	Fellahin	Northern Africa	33	1945-1951
11	Fon	Western Africa	34	1920-1931
12	Ganda	Eastern Africa	35	1932-1932
13	Garo	South Asia	36–38	1908-1965
14	Gikuyu	Eastern Africa	39–41	1905-1994
15	Gond	South Asia	42	1951-1959
16	Gusii	Eastern Africa	43, 44	1982-1985
17	Hausa	Western Africa	45, 46	1940-1967
18	Ila	Southern Africa	47–49	1902-1914
19	Inner Mongolia	Central Asia	50, 51	1988-1990
20	Iran	Middle East	52	1930-1984
21	Kaffa	Eastern Africa	53	No date
22	Kanuri	Western Africa	54, 55	1956-1965
23	Kapsiki	Western Africa	30	1978-1991
24	Katab	Western Africa	56	1980-1984
25	Khasi	South Asia	57, 58	1955-1956
26	Konso	Eastern Africa	59–61	1965-1997
27	Korea	East Asia	62, 63	1975-1976
28	Lepcha	Central Asia	64–67	1937-1984
29	Lozi	Southern Africa	68–72	1940-1952
30	Manchu	East Asia	73	No date
31	Miao	East Asia	74, 75	1980-199
32	Mossi	Western Africa	76–78	1908-1956
33	Ngibelai Turkana	Eastern Africa	79–81	1948-1996
34	Ngikebotok Turkana	Eastern Africa	79–81	1948-1996
35	Northern Tuareg	Northern Africa	82–84	1929-1962
36	Nuba	Eastern Africa	85	1966-1980
37	Nuer	Eastern Africa	23, 86–88	1930-1944
38	Nupe	Western Africa	89	1934-1936
39	Nyakyusa	Eastern Africa	90–92	1934-1938
40	Pashtun	Central Asia	93	1954-1979
41	Rwandans	Central Africa	94, 95	1907-1925
42	Santal	South Asia	96	1931-1945
43	Sherpa	Central Asia	97, 98	1979-1987
44	Shilluk	Eastern Africa	99	1940-1954
45	Shluh	Northern Africa	100	1950-1970
46	Shona	Southern Africa	101–103	1945-1948
47	Somali	Eastern Africa	104–107	1955-1970
48	Songhai	Western Africa	108	1970-1987
49	Southern Tuareg	Northern Africa	83, 84	1951-1962
50	Tallensi	Western Africa	109–111	1934-1945
51	Tamil	South Asia	112–115	1949-1961

Continuation of Table S3				
**ID**	**Culture**	**Region**	**References**	**Field dates**
52	Teda	Central Africa	116, 117	1930-1955
53	Telugu	South Asia	118	1970-1972
54	Tiv	Western Africa	119–125	1916-1953
55	Tonga	Southern Africa	126–131	1949-2004
56	Tsonga	Southern Africa	132	1895-1909
57	Wolof	Western Africa	133–136	1843-1957

Supplementary Figures

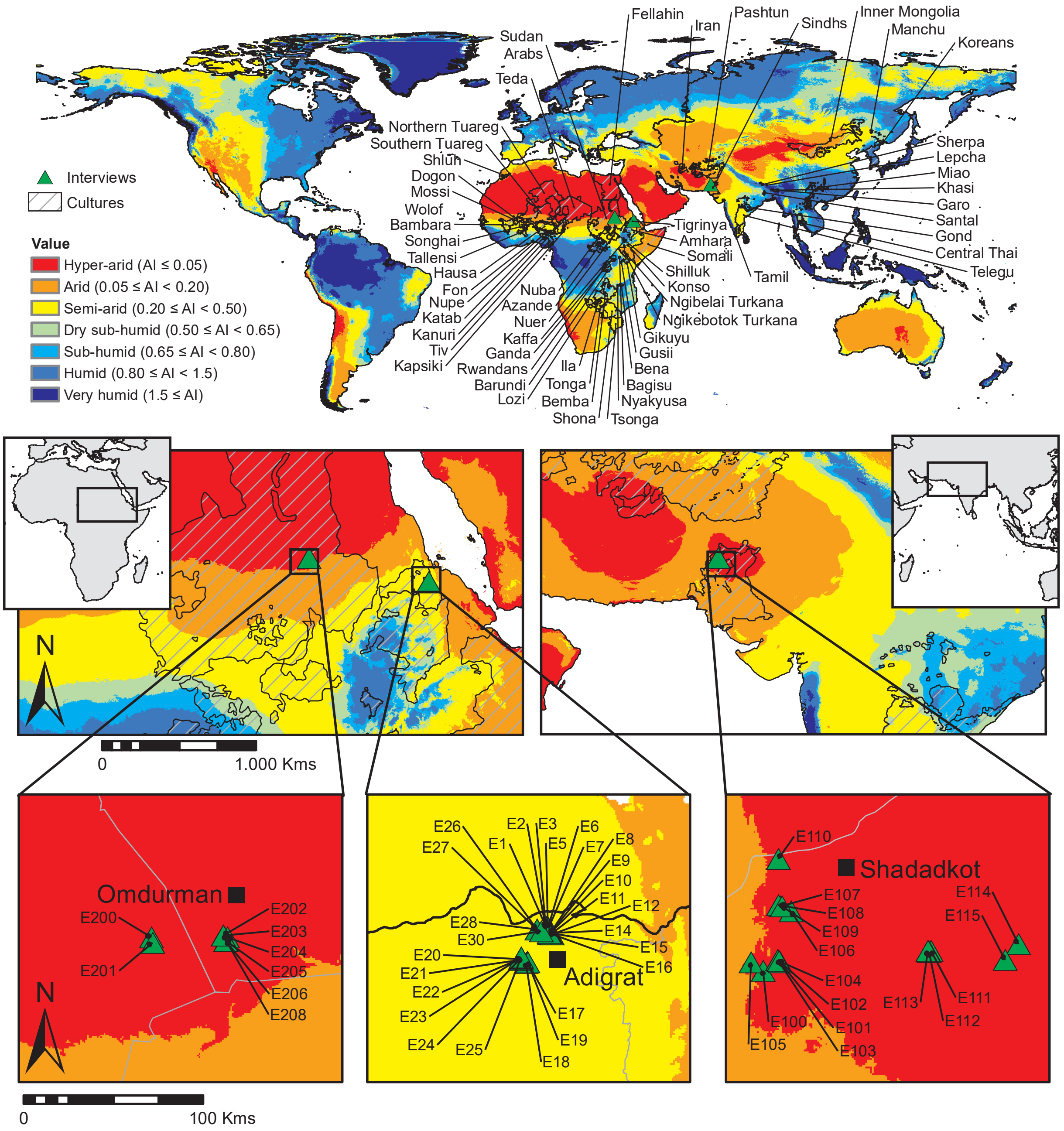

Figure 1. Figure S1. World map displaying the location of all cultures included in the study. At the bottom, zoom-ins of the locations of ethnographic fieldwork with position of all the interviews performed.

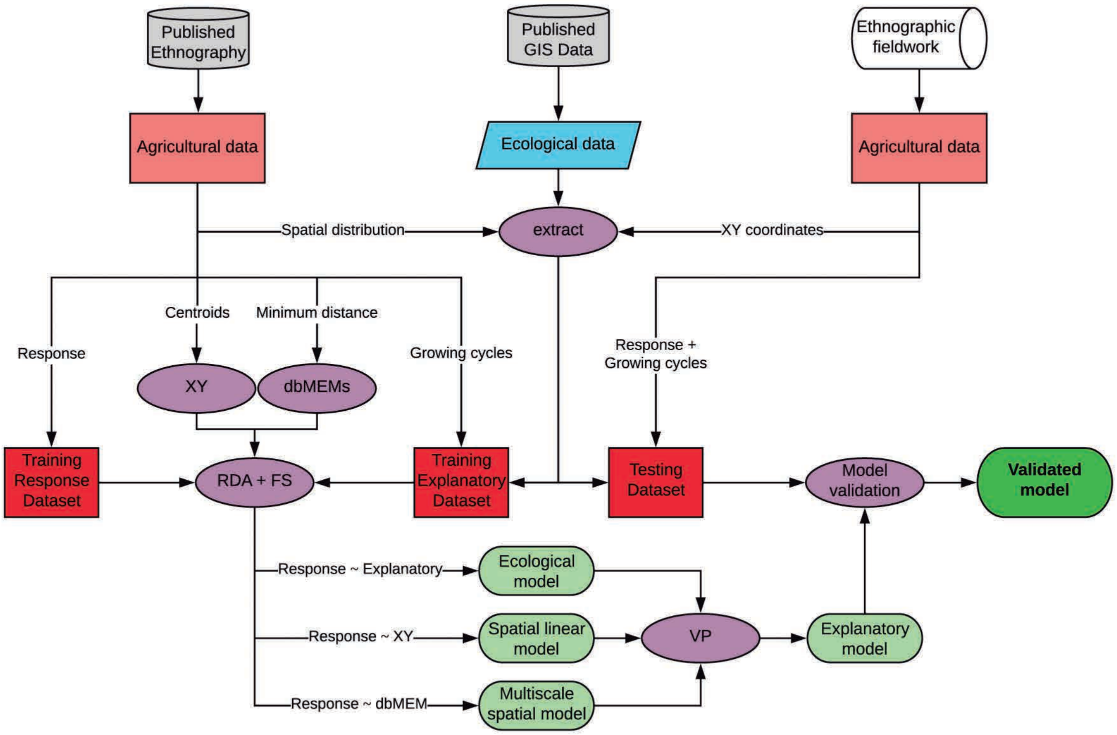

Figure 2. Figure S2. Reduced version of the workflow used in this study. Grey boxes represent online databases. Blue boxes contain spatial data. Red rectangles correspond to datasets. Processing data steps in R are purple ovals. Green boxes represent models

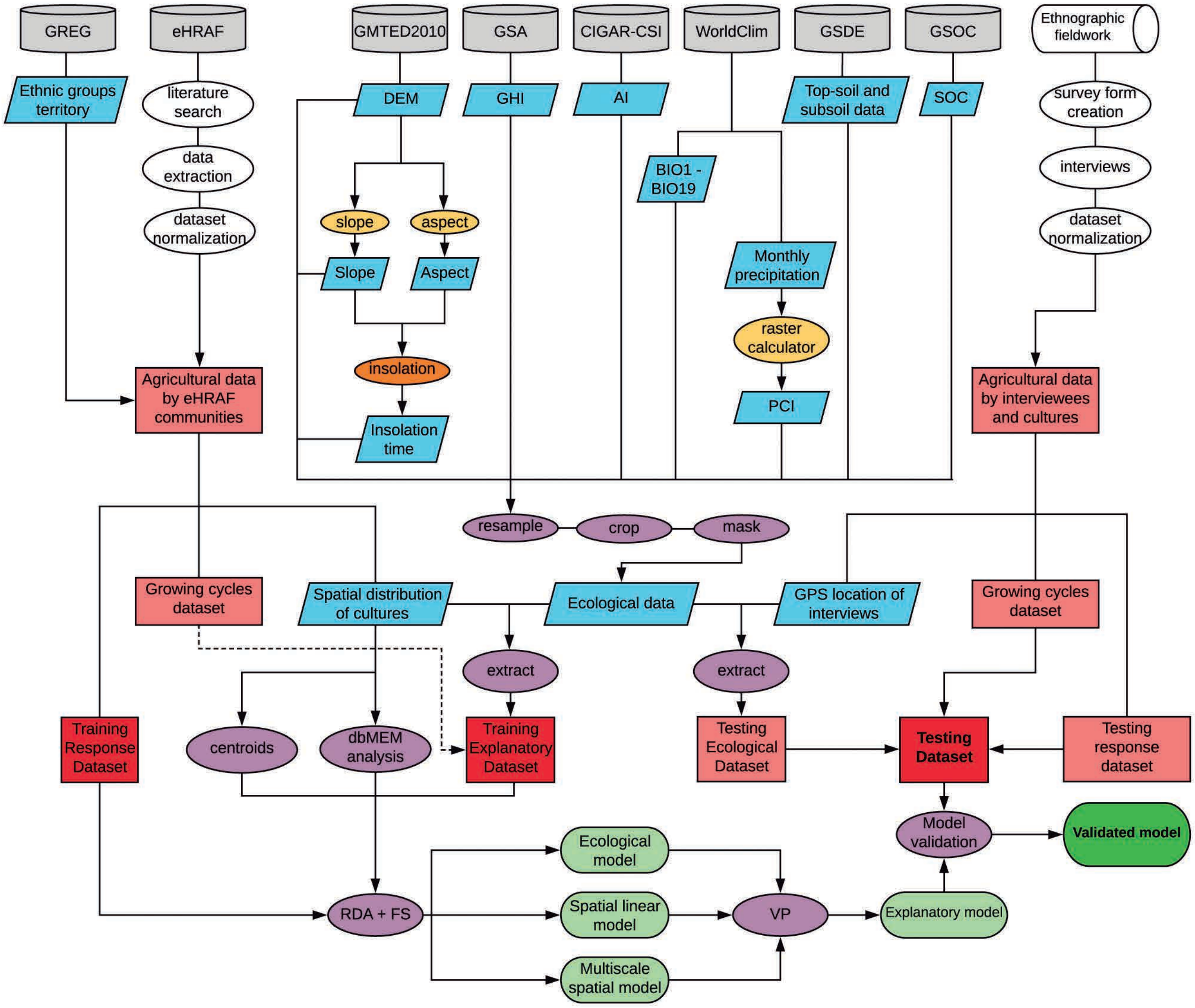

Figure 3. Figure S3. Full workflow of the data gathering and analysis used in this study. Grey boxes represent online databases. White ovals represent data processing. Yellow ovals represent ArcGIS 10.6. processing. Orange ovals represent QGIS 3.4.15 with GRASS 7.8.2 processing. Blue boxes contain spatial data. Red rectangles correspond to datasets. Processing data steps in R are purple ovals. Green boxes represent models.

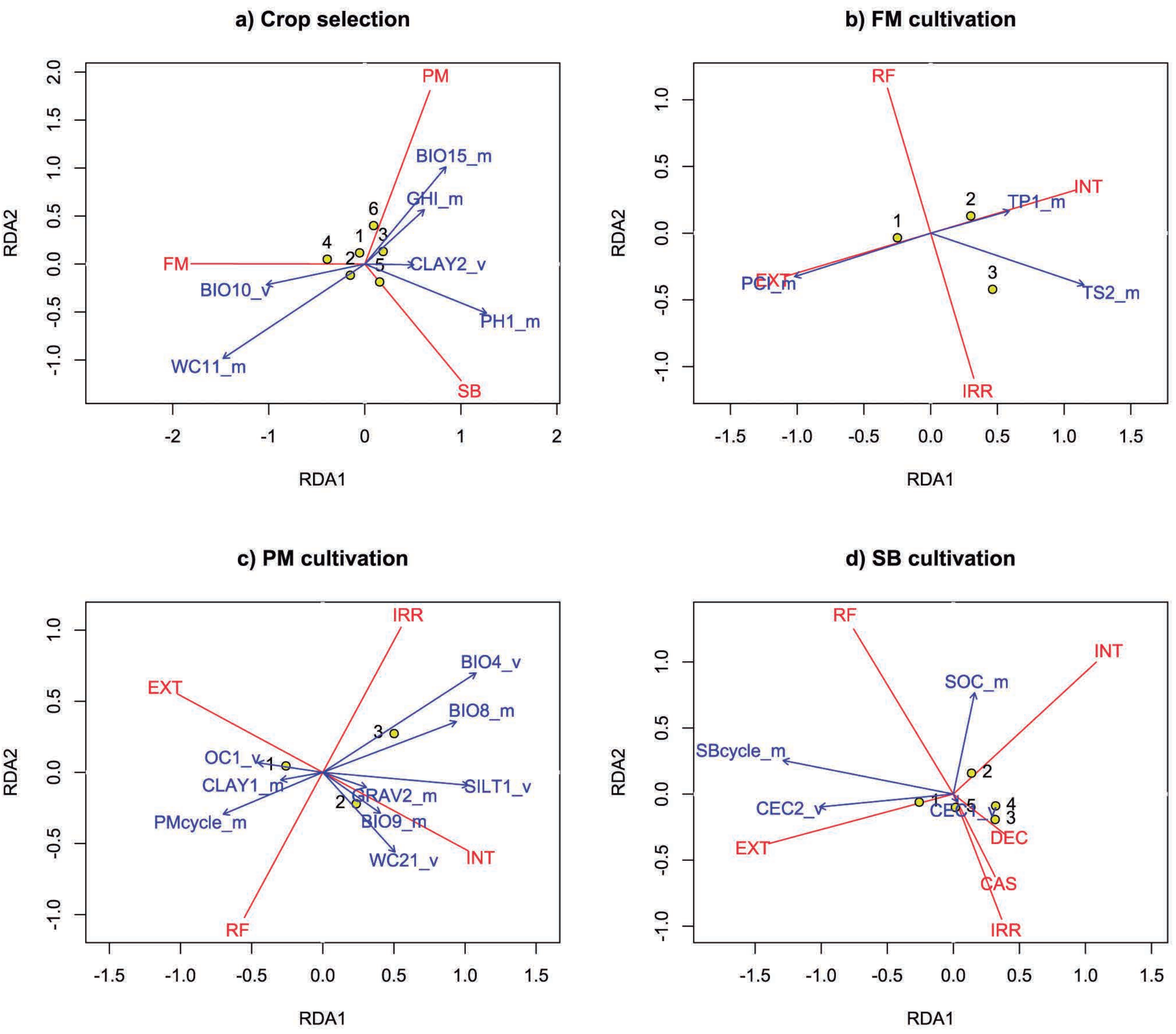

Figure 4. Figure S4. Triplots of RDA by forward selection variables for: a) Crop selection (1 = FM-PM-SB, 2 = FM-SB, 3 = PM-SB, 4 = FM, 5 = SB, 6 = PM); b) finger millet cultivation (1 = EXT-RF, 2 = INT-RF, 3 = INT-IRR); c) PM cultivation (1 = EXT-RF, 2 = INT-RF, 3 = INT-IRR); d) SB cultivation (1 = EXT-RF, 2 = INT-RF, 3 = INT-IRR, 4 = INT-DEC, 5 = CAS-RF). EXT=Extensive; INT=Intensive; RF=Rainfed; IRR=Irrigated; CAS=Casual agriculture; DEC=*Décrue* agriculture. For variable coding, see Table S3 in Supplementary Information

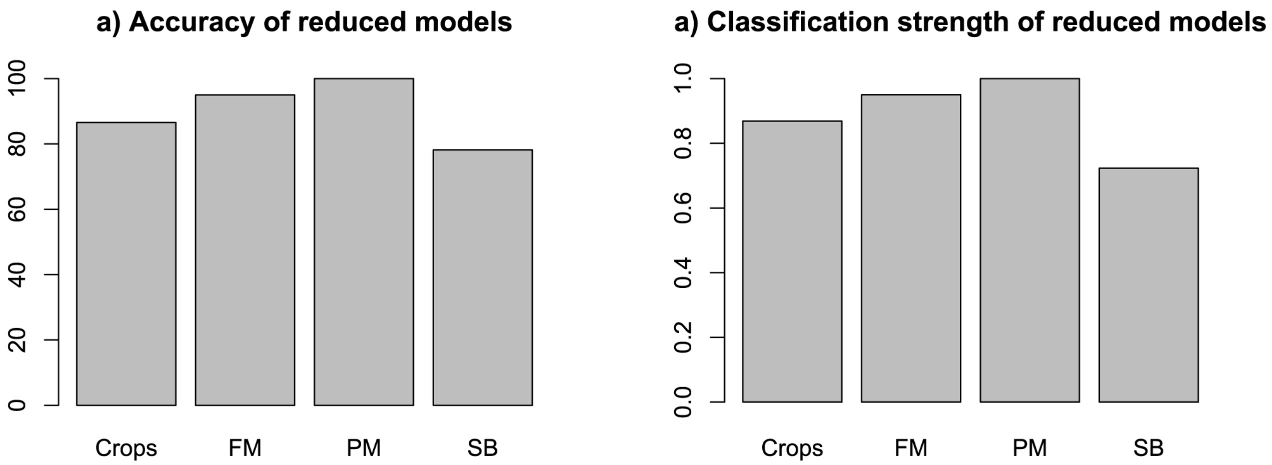

Figure 5. Figure S5. Barcharts of accuracy and classification strength (F1-score) by model.

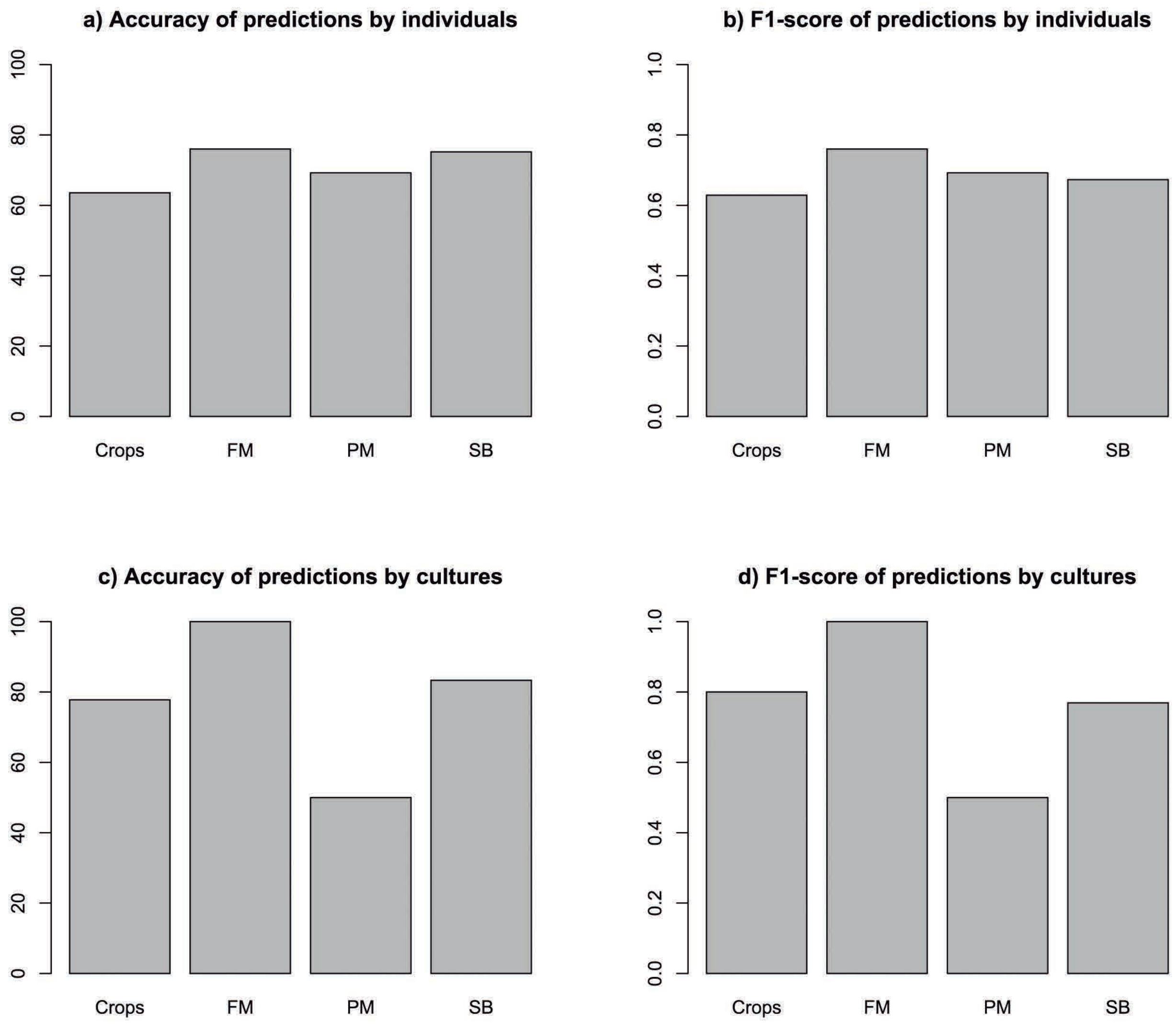

Figure 6. Figure S6. Barcharts of model's predictions accuracy and F1-score by individuals (a, b) and cultures (c, d).

References

1. Murdock, G. *Atlas of world cultures.* (University of Pittsburgh Press, Pittsburgh, 1981).
2. Lancelotti, C., Biagetti, S., Zerboni, A., Usai, D. & Madella, M. The archaeology and ethnoarchaeology of rain-fed cultivation in arid and hyper-arid North Africa. *Antiquity* **93**, 1026–1039, DOI: 10.15184/aqy.2019.109 (2019).
3. Danielson, J. J. & Gesch, D. B. *Global multi-resolution terrain elevation data 2010 (GMTED2010)* (US Geological Survey Open-File Report 2011-1073, Washington D.C., 2011).
4. Estima, J., Fichaux, N., Menard, L. & Ghedira, H. The global solar and wind atlas. In *Proceedings of the 1st ACM SIGSPATIAL International Workshop on MapInteraction - MapInteract '13*, 36–39, DOI: 10.1145/2534931.2534933 (ACM Press, New York, New York, USA, 2013).
5. Zomer, R. J., Trabucco, A., Bossio, D. A. & Verchot, L. V. Climate change mitigation: A spatial analysis of global land suitability for clean development mechanism afforestation and reforestation. *Agric. Ecosyst. & Environ.* **126**, 67–80, DOI: 10.1016/j.agee.2008.01.014 (2008).
6. Fick, S. E. & Hijmans, R. J. WorldClim 2: new 1-km spatial resolution climate surfaces for global land areas. *Int. J. Climatol.* **37**, 4302–4315, DOI: 10.1002/joc.5086 (2017).
7. Hiederer, R. & Köchy, M. Global soil organic carbon estimates and the harmonized world soil database. *EUR* **79**, 25225 (2011).
8. Shangguan, W., Dai, Y., Duan, Q., Liu, B. & Yuan, H. A global soil data set for earth system modeling. *J. Adv. Model. Earth Syst.* **6**, 249–263, DOI: 10.1002/2013MS000293 (2014).
9. Messing, S. D. *The highland-plateau Amhara of Ethiopia*. Ph.D. thesis, University of Pennsylvania (1957).
10. Hoben, A. *Land tenure among the Amhara of Ethiopia; the dynamics of cognatic descent* (University of Chicago Press, Chicago, 1973).
11. Anderson, R. G. *Some tribal customs in their relation to medicine and morals of the nyam-nyam and gour people inhabiting the eastern Bahr-el-Ghazal* (Bailier, Tindall and Cox, London, 1911).
12. Larken, P. M. An account of the Zande. *Sudan notes records* **9**, 1–56 (1926).
13. Culwick, G. M. *A dietary survey among the Zande of the South-Western Sudan* (Ministry of Agriculture, Sudan Government, Khartoum, 1950).
14. Baxter, P. T. W. & Butt, A. *The Azande and Related Peoples of the Anglo-Egyptian Sudan and Belgian Congo* (Routledge, London, 1953).
15. Schlippe, P. D. *Shifting cultivation in Africa: the Zande system of agriculture* (Routledge, London, 1956).
16. Heald, S. *Controlling anger: the sociology of {Gisu} violence* (Manchester University Press, Manchester, 1989).
17. Paques, V. *Les Bambara* (Presses Universitaires de France, Paris, 1954).
18. Toulmin, C. *Cattle, women and wells: managing household survival in the Sahel* (Oxford University Press, Oxford, 1992).
19. Becker, L. C. Access to labor in rural Mali. *Hum. Organ.* **55**, 279–288 (1996).
20. Becker, L. C. Garden money buys grain: food procurement patterns in a malian village. *Hum. Ecol.* **28**, 219–250 (2000).
21. Meyer, H. *Die Barundi, Eine völkerkunlich Studie aus Deutsch-Ostafrika* (Ott Spamer, Leipzig, 1959).
22. Albert, E. Women of Burundi: A Study of Social Values. In Paulme, D. (ed.) *Women of Tropical Africa*, 179–217 (Routledge and Kegan Paul, London, 1963).
23. Evans-Pritchard, E. E. Economic life of the Nuer: cattle. *Sudan Notes Rec.* **21**, 31–37 (1938).
24. Richards, A. I. *Chisungu: a girls' initiation ceremony among the Bemba of Northern Rhodesia* (Faber & Faber, London, 1956).
25. Lagacé, R. O. & Skoggard, I. A. *Culture Summary: Bemba* (Human Relations Area File, New Haven, 1997).
26. Culwick, A. T., Culwick, M. G. & Kiwanga, T. *Ubena of the Rivers* (G. Allen & Unwin Limited, London, 1935).
27. Judd, P. *Irrigated agriculture in the Central Plain of Thailand* (Australian National University, Canberra, 1973).
28. Paulme, D. & Schützw, F. *Social organization of the Dogon (French Sudan)* (Edition Domat Montchrestien, Paris, 1940).
29. Griaule, M. & Dieterlen, G. *The pale fox* (Afrikan World Books, Baltimore, 1986).

30. Van Beek, W. *Harmony vs. autonomy: models of agricultural fertility among the Dogon and the Kapsiki* (Uppsala University Press, Uppsala, 1991).

31. Van Beek, W. Processes and limitations of Dogon agricultural knowledge. In Hobart, M. (ed.) *An anthropological critique of development*, 55–82 (Routledge, London, 2002).

32. Griaule, M. *Masques dogons* (1994).

33. Amraar, H. *An Egyptian Village Growing Up: Silwa, the Governorate of Aswa*. Ph.D. thesis, University of Florida, Gainesville (1988).

34. Herskovits, M. *Dahomey: an ancient west African kingdom* (J. J. Augustin, New York, 1938).

35. Mair, L. P. *An African People in the Twentieth Century* (Routledge, London, 1965).

36. Playfair, A. *The Garos* (David Nutt, London, 1909).

37. Burling, R. *Rengsanggri: family and kinship in a Garo village* (University of Pennsylvania Press, Pennsylvania, 1963).

38. Majumdar, D. N. *Culture change in two Garo villages* (Anthropological Survey of India, Calcutta, 1978).

39. Kenyatta, J. *Facing Mount Kenya: The Tribal Life of the Gikuyu (1938)* (Secker and Warburg, London, 1953).

40. Routledge, W. S. & Routledge, K. *With a prehistoric people: the Akikuyu of British East Africa* (Psychology Press Ltd, London, 1968).

41. Davison, J. *Voices from Mutira: Change in the lives of rural Gikuyo women, 1910-1995* (Lynne Rienner Publishers, 1996).

42. Fuchs, S. *The Gond and Bhumia of Eastern Mandla* (Asia Publishing House, London, 1960).

43. Hakansson, T. *Bridewealth, women and land: Social change among the Gusii of Kenya* (Uppsala University Press, Uppsala, 1990).

44. Hakansson, N. T. Grain, cattle, and power: social processes of intensive cultivation and exchange in precolonial western Kenya. *J. Anthropol. Res.* **50**, 249–276 (1994).

45. Forde, C. D. & Scott, R. Native Economies of Nigeria: Being the First Volume of a Study of the Economics of a Tropical Dependency. (1946).

46. Hill, P. *Rural Hausa: a village and a setting* (Cambridge University Press, Cambridge, 1972).

47. Smith, E. W. & Dale, A. M. *The Ila-speaking peoples of northern Rhodesia* (MacMillan, London, 1920).

48. Jaspan, M. A. *The Ila-Tonga Peoples of North-Western Rhodesia: West Central Africa* (Routledge, London, 1953).

49. Fielder, R. J. Economic spheres in pre-colonial Ila society. *Afr. Soc. Res.* **28**, 617–641 (1979).

50. Chang, Y. T. *The Economic Development and Prospects of Inner Mongolia (Chahar, Suiyuan, and Ningsia)* (Commercial Press Limited, Shanghai, 1933).

51. Pasternak, B. & Salaff, J. W. *Cowboys and Cultivators: The Chinese Of Inner Mongolia* (Routledge, London, 1993).

52. Hooper, D., McNair, J. B. & Field, H. *Useful plants and drugs of Iran and Iraq* (Field Museum of Natural History, Chicago, 1937).

53. Adem, T. A. *Culture summary: Kaffa* (Human Relations Area Files, New Haven, 2012).

54. Cohen, R. *The Kanuri of Bornu* (Holt, Rinehart & Winston, New York, 1967).

55. Rosman, A. *Social structure and acculturation among the Kanuri of northern Nigeria* (University Microfilms International, Ann Arbor, 1978).

56. Bonat, Z. A. Aspects of the economic and social history of the atyab (katab) c. 1800-1960 ad. *Savanna: A J. Environ. Soc. Sci.* **10**, 39–57 (1989).

57. Gurdon, P. R. T. *Khasis* (Macmillan and Co., London, 1907).

58. Nakane, C. *Garo And Khasi: A Comparative Study in Matrilineal Systems* (Mouton De Gruyter, Berlin, 1967).

59. Hallpike, C. R. Konso agriculture. *J. Ethiop. Stud.* **8**, 31–43 (1970).

60. Hallpike, C. R. *Konso Of Ethiopia: A Study of the Values of an East Cushitic People* (Oxford University Press, Oxford, 2008).

61. Hallpike, C. R. *Culture summary: Konso* (Human Relations Area Files, New Haven, 2016).

62. Han, C. C. *Social organization of an upper Han hamlet in Korea* (1949).

63. Chun, K.-s. & Chŏn, K.-s. *Reciprocity and Korean society: An ethnography of Hasami*. 6 (Seoul National University Press, 1984).

64. Gorer, G. & Hutton, J. H. *Himalayan village: an account of the Lepchas of Sikkim* (Michael Joseph Ltd, London, 1938).

65. Morris, J. *Living with Lepchas: a book about the Sikkim Himalayas* (Heinemann Limited, Portsmouth, 1938).

66. Siiger, H. & Rischel, J. *The Lepchas: Culture and religion of a Himalayan people* (National Museum of Denmark, Copenhagen, 1967).

67. Foning, A. R. *Lepcha, my vanishing tribe* (Sterling Publishers, New Delhi, 1987).

68. Gluckman, M. *Economy of the central Barotse plain* (Rhodes-Livingstone Institute, Livingstone, 1941).

69. Gluckman, M. *Essays on Lozi land and royal property* (Rhodes-Livingstone Institute, Livingstone, 1943).

70. Gluckman, M. *The Lozi of Barotseland in north-western Rhodesia* (Manchester University Press, Manchester, 1951).

71. Peters, D. U. *Land usage in Barotseland* (Rhodes-Livingstone Institute, Livingstone, 1960).

72. Beierle, J. *Culture summary: Lozi* (Human Relations Area Files, New Haven, 1995).

73. Isett, C. M. *State, peasant, and merchant in Qing Manchuria, 1644-1862* (Stanford University Press, Stanford, 2007).

74. Diamond, N. Ethnicity and the state: the Hua Miao of Southwest China. In Toland, J. B. (ed.) *Ethnicity and the State*, 55–78 (Transaction Publishers, New Brunswick, 1993).

75. Diamond, N. *Culture Summary: Miao* (Human Relations Area Files, New Haven, 2009).

76. Tauxier, L. & Brunel, A. *The black population of the Sudan, Mossi and Gourounsi country, documents and analyses.* (Emile Larose, Librairie-Editeur, Paris, 1912).

77. Hammond, P. B. *Economic change and Mossi acculturation* (University of Chicago Press, Chicago, 1959).

78. Mangin, E. *The Mossi: Essay on the Manners and Customs of the Mossi People in the Western Sudan* (Human Relations Area File, New Haven, 1959).

79. Gulliver, P. H. *A preliminary survey of the Turkana: A report compiled for the Government of Kenya* (University of Cape Town, Cape Town, 1951).

80. McCabe, J. T. & Dyson-Hudson, R. *South Turkana Nomadism: Coping with an Unpredictably Varying Environment* (HRAFlex Books, New Haven, 1985).

81. Bollig, M. Turkana Herders of the Dry Savanna. *Ecol. Biobehav. Response Nomads to an Uncertain Environ. Am. J. Hum. Biol.* **13**, 81–91 (2001).

82. Lhote, H. *The Hoggar Tuareg* (Human Relations Area Files, New Haven, 1944).

83. Nicolaisen, J. *Political systems of pastoral Tuareg in Air and Ahagga* (Harper and Row, New York, 1959).

84. Nicolaisen, J. *Ecology and culture of the pastoral Tuareg: with particular reference to the Tuareg of Ahaggar and Ayr* (The National Museum of Copenhagen, Copenhagen, 1963).

85. Faris, J. C. *Southeast Nuba social relations* (Alano Edition Herodot, Aachen, 1989).

86. Evans-Pritchard, E. E. *The Nuer: A description of the modes of livelihood and political institutions of a Nilotic people* (Oxford University Press, London, 1940).

87. Butt, A. *The Nilotes of the Anglo-Egyptian Sudan and Uganda* (International African Institute, London, 1952).

88. Howell, P. P. *A manual of Nuer law: Being an account of customary law, its evolution and development in the courts established by the Sudan Government* (Routledge, London, 1954).

89. Nadel, S. F. *A Black Byzantium: The Kingdom of Nupe in Nigeria* (International Institute of African Languages and Cultures, Oxford, 1942).

90. Wilson, G. *Land Rights of Individuals Among the Nyakyusa* (Rhodes-Livingstone Institute, Livingstone, 1938).

91. Wilson, M. H. *For Men and Elders: Change in the Relations of Generations of Men and Women among the Nyakyusa-Ngonde People, 1875-1971.* (International African Institute, London, 1977).

92. Kalinga, O. J. Towards a Better Understanding of Socio-Economic Change in 18th-and 19th-Century Ungonde. *Cahiers d'Études africaines* **93**, 87–100 (1984).

93. Barth, F. *Features of person and society in Swat: Collected essays on Pathans* (Routledge, London, 1981).

94. Czekanowski, J. *Investigations in the area between the Nile and the Congo* (Human Relations Area Files, New Haven, 1959).

95. Pagès, F. G. *A Hamitic Kingdom in the Center of Africa in Ruanda on the Shores of Lake Kivu (Belgian Congo): Un Royaume Hamite Au Centre de L'Afrique. Au Ruanda Sur Les Bords Du Lac Kivu (Congo Belge)* (Human Relations Area Files, 1960).

96. Biswas, P. C. *Santals of the Santal Parganas* (Bharatiya Adimjati Sevak Sangh, Delhi, 1956).

97. Kunwar, R. *Fire of Himal. An Anthropological Study of the Sherpas of Nepal Himalayan Region* (Nirala, Delhi, 1989).

98. Stevens, S. F. *Sherpa settlement and subsistence: cultural ecology and history in highland Nepal* (1990).

99. Dempsey, J. *Mission on the Nile* (Burns & Oates, London, 1956).

100. Berque, J. *Social structures of the High Atlas* (Presses Universitaires de France, Paris, 1955).

101. Kuper, H., Hughes, A. J. B. & Van Velsen, J. *The Shona and Ndebele of Southern Rhodesia: Southern Africa* (Routledge, London, 1954).

102. Holleman, J. F. *Shona customary law: with reference to kinship, marriage, the family and the state* (Manchester University Press, Manchester, 1969).

103. Bhila, H. H. *Trade and Politics in a Shona Kingdom: the Manyika and their African and Portuguese neighbours, 1575-1902* (Longman, London, 1982).

104. Lewis, I. M. *Marriage and the family in Northern Somaliland* (East African Institute of Social Research, Kampala, 1962).

105. Galaal, M. H. *The Terminology and Practice of Somali Weather Lore, Astronomy, and Astrology* (Original publisher Mogadishu, Mogadishu, 1968).

106. Helander, B. & Beierle, J. *Culture summary: Somali* (Human Relations Area Files, New Haven, 1997).

107. Lewis, I. M. & Samatar, S. S. *A Pastoral Democracy: a study of pastoralism and politics among the northern Somali of the Horn of Africa* (International African Institute, London, 1999).

108. Stoller, P. *Fusion of the worlds: An ethnography of possession among the Songhay of Niger* (University of Chicago Press, Chicago, 1989).

109. Fortes, M. & Fortes, S. L. Food in the domestic economy of the Tallensi. *Africa* **9**, 237–276 (1936).

110. Fortes, M. Communal fishing and fishing magic in the Northern Territories of the Gold Coast. *The J. Royal Anthropol. Inst. Gt. Br. Irel.* **67**, 131–142 (1937).

111. Fortes, M. *The dynamics of clanship among the Tallensi: Being the first part of an analysis of the social structure of a Trans-Volta tribe* (Routledge, London, 1945).

112. Sivertsen, D. *When caste barriers fall. A study of social and economic change in a south Indian village* (Scandinavian University Humanities Press, New York, 1963).

113. Nambiar, P. K. *Census of India 1961* (Manager of Publication, Delhi, 1965).

114. Haswell, M. R. *Economics of development in village India* (Taylor & Francis, London, 1967).

115. Dumont, L. *A Sub-caste of South India: Social Organization and Religion of the Pramalai Kallar* (Human Relations Area Files, New Haven, 1983).

116. Kronenberg, A. *Die Teda von Tibesti* (Verlag Ferdinand Berger, Vienna, 1958).

117. Chapelle, J. *Black nomads of the Sahara* (Human Relations Area Files, New Haven, 1982).

118. Tapper, B. *Rivalry and tribute: Society and ritual in a Telugu village in south India* (South Asia Books, London, 1988).

119. Abraham, R. C. *The Tiv People* (The Government Printer, Lagos, 1933).

120. East, R. M. *Akiga's Story: the Tiv tribe as seen by one of its members* (Oxford University Press, London, 1939).

121. Bohannan, P. & Bohannan, L. *The Tiv of Central Nigeria* (International African Institute, London, 1953).

122. Bohannan, P. Concepts of time among the Tiv of Nigeria. *Southwest. J. Anthropol.* **9**, 251–262 (1953).

123. Bohannan, P. *Tiv Farm and Settlement* (Her Majesty's Stationery Office, London, 1957).

124. Bohannan, P. *Three source notebooks in Tiv ethnography* (Human Relations Area Files, New Haven, 1966).

125. Bohannan, P. & Bohannan, L. *Tiv economy* (Northwestern University Press, Evanston, 1968).

126. Scudder, T. *The ecology of the Gwembe Tonga* (Manchester University Press, Manchester, 1962).

127. Scudder, T. *Gathering among African woodland savannah cultivators* (University of Zambia Institute, Lusaka, 1971).

128. Scudder, T. *Ecological bottlenecks and the development of the Kariba Lake basin* (Tom Stacey, London, 1972).

129. Colson, E. Land law and land holdings among Valley Tonga of Zambia. *J. Anthropol. Res.* **42**, 261–268 (1986).

130. Reynolds, B. *The material culture of the peoples of the Gwembe Valley* (Praeger, New York, 1968).

131. Cliggett, L. *Grains from Grass: Aging, Gender, and Famine in Rural Africa* (Cornell University Press, Ithaca, 2005).

132. Junod, H. A. *The life of a South African tribe* (Neuchâtel Impr. Attinger, Hauterive, 1927).

133. Boilat, A. P. *Senegalese Sketches* (P. Bertrand Libraire-Editeur, Paris, 1853).

134. Audiger, J. & Moore, G. W. *Wolof of the Bas-Ferlo* (L'Institut de Géographie de la Faculté des Lettres de Bordeaux, Bordeaux, 1961).

135. Gamble, D. P. *The Wolof of Senegambia: together with notes on the Lebu and the Serer* (International African Institute, London, 1967).

136. Venema, L. B. *The Wolof of Saloum: social structure and rural development in Senegal* (Centre for Agricultural Publishing and Documentation, Wageningen, 1978).
