# Supplementary material for: Small-scale farming in drylands: New models for resilient practices of millet and sorghum cultivation
Source: PLoS One. 2023 Feb 2;18(2):e0268120. doi: 10.1371/journal.pone.0268120 (PMC9894398; doi:10.1371/journal.pone.0268120)
Supplement: Attachment — Submitted filename: Response to reviewers.pdf [file pone.0268120.s002.pdf]

PONE-D-21-06936
Small-scale farming in drylands: New models for resilient practices of millet and sorghum cultivation
PLOS ONE

Additional Editor Comments (if provided):

Please provide additional data as suggested by reviewer 2

RE. Reviewer 2 does not ask for additional data, so we are not sure what this comment refers to. If the editor refers to reviewer #1's comments, please see our answers below.

Reviewers' comments:

Reviewer's Responses to Questions

Comments to the Author

1. Is the manuscript technically sound, and do the data support the conclusions?

The manuscript must describe a technically sound piece of scientific research with data that supports the conclusions. Experiments must have been conducted rigorously, with appropriate controls, replication, and sample sizes. The conclusions must be drawn appropriately based on the data presented.

Reviewer #1: No

Reviewer #2: Partly

RE. We do not understand how the reviewers have reached this decision considering that they both state in their review, and in response to question 2 below, that they are not able to assess the analysis or evaluate the data. Consequently, in our opinion, they should not be able to evaluate whether the data support the conclusion or if the experiments (in this case modelling experiments) have been conducted rigorously.

---

2. Has the statistical analysis been performed appropriately and rigorously?

Reviewer #1: I Don't Know

Reviewer #2: I Don't Know

RE. Considering that this is a modelling paper heavily based on statistics, this answer alone should disqualify the reviews as experts who can provide informed assessments.

---

3. Have the authors made all data underlying the findings in their manuscript fully available?

The PLOS Data policy requires authors to make all data underlying the findings described in their manuscript fully available without restriction, with rare exception (please refer to the Data Availability Statement in the manuscript PDF file). The data should be provided as part of the manuscript or its supporting information, or deposited to a public repository. For example, in addition to summary statistics, the data points behind means, medians and variance measures should be available. If there are restrictions on publicly sharing data—e.g. participant privacy or use of data from a third party—those must be specified.

Reviewer #1: Yes

Reviewer #2: Yes

---

4. Is the manuscript presented in an intelligible fashion and written in standard English?

PLOS ONE does not copyedit accepted manuscripts, so the language in submitted articles must be clear, correct, and unambiguous. Any typographical or grammatical errors should be corrected at revision, so please note any specific errors here.

Reviewer #1: Yes

Reviewer #2: Yes

---

5. Review Comments to the Author

Please use the space provided to explain your answers to the questions above. You may also include additional comments for the author, including concerns about dual publication, research ethics, or publication ethics. (Please upload your review as an attachment if it exceeds 20,000 characters)

Reviewer #1: My problem with this paper lies in the fact that I cannot discern why the work was done. The Introduction doesn't describe a problem to be addressed nor a hypothesis that could be tested.

RE. We appreciate the fact that the aims of the papers might not be sufficiently clear and we are happy to clarify them if needed. However, we state what is the purpose of the paper in the introduction. Copied from Introduction:

Our work contributes to this debate [i.e. the recognised inconsistency between academic and traditional knowledge in agricultural policies] *by providing a global assessment of, and novel data on traditional practices adopted by small-scale farmers. Differently from the*

yield-oriented models normally developed in agronomic studies, we focus on the techniques that have been traditionally applied to cultivate these crops by small-scale farmers. We integrate traditional ecological knowledge (TEK) with academic ecological knowledge (AEK) in order to create models that aim at understanding how traditional agricultural systems relate to the surrounding environment. (Introduction, page 2 lines 12-19)

The results themselves are poorly described – what is the function of Fig 1 and what does Fig 2 represent in real terms?

RE. Figure 1 is a map showing the location of the data points. Figure 2 is a Venn diagram showing variation partition analysis results. Clearly the reviewer cannot understand the figures (although Figure 1 being a map should be pretty self-explanatory) as he/she does not understand the analysis that is at the core of the whole paper. We also provide extended results in the appendix, with much detail on the statistical analysis, which the reviewer has probably not even looked at considering his/her inability to understand the statistics.

Consequently, the Discussion and Conclusions also fail to reveal what all the work was about, other than to make another plea for TEK to be taken into account in agricultural research and development. I have no problem with that at all but this paper doesn't add anything useful to support that plea.

RE. Again, if the reviewer does not understand the paper, he/she cannot understand whether the conclusions are sound and novel.

At the outset the authors acknowledge that it is not just the ecological and geographical variables that determine crop choice by farmers, but then go on to exclude any consideration of socio-economic, markets, taste preferences, cultural or historical issues.

RE. In all modelling papers some variables are excluded from the analysis. The choice of which variables to retain and which to exclude is fully justified in the paper:

Even though agricultural activities can be influenced by several factors, […]-which all contribute to the high variability of agricultural systems [16]- we consider that, under specific environmental and cultural contexts, societies can only adopt a finite number of agricultural solutions. However, as it has been shown that ecological or medical reasons are often at the root of food preferences, even in their more extreme form of food taboos [17], in this work we concentrate on the ecological drivers rather than the cultural background of cultivation practices. (Introduction, pages 3-3. Lines 25-33)

There are very many reasons why farmers grow the crops they do and some reasons may have nothing to do with how well the crops are suited to the physical conditions. A classic case is that of farmers in the drier regions of Southern Africa (Botswana and southern Zimbabwe) who know that maize will fail often but plant it every year anyway (often citing, amongst other reasons, the labour savings in birdscaring to protect the 'naked' seeds of sorghum and pearl millet). In a related issue, the data used in this paper do not appear (I may be wrong) to include information on whether the crops chosen actually grow very well. Apart from many other possible reasons for choosing them it may be that other crops fare even

worse. Or they used to grow well historically but the conditions have worsened? I could go on and on.

RE. Indeed, the list of reasons why people decide to cultivate some crops and not others is probably limitless. That is why, when using a model-based approach to try and pinpoint the general mechanisms behind these decisions, some variables (such as cultural decisions) cannot be taken into consideration.

I do not believe this manuscript is worthy of publication in PLOS One.

RE. In our opinion, and considering what has been detailed above, no sound reason has been given on why this decision was reached, especially considering the poor understanding of the modelling approach used in this paper.

Reviewer #2: Unable to assess the analysis. The text is well written and clear. Nothing more to addUnable to assess the analysis. The text is well written and clear. Nothing more to addUnable to assess the analysis.

RE. We do not really have any response to this reviewer rather than reiterate the fact that if he/she is unable to assess the analysis, he/she is not qualified to be a reviewer of this paper. Nonetheless we appreciate the fact that he/she considers the paper well written and clear.

---

6. PLOS authors have the option to publish the peer review history of their article (what does this mean?). If published, this will include your full peer review and any attached files.

If you choose "no", your identity will remain anonymous but your review may still be made public.

Do you want your identity to be public for this peer review? For information about this choice, including consent withdrawal, please see our Privacy Policy.

Reviewer #1: No

Reviewer #2: No
