# Supplementary material for: Small-scale farming in drylands: New models for resilient practices of millet and sorghum cultivation
Source: PLoS One. 2023 Feb 2;18(2):e0268120. doi: 10.1371/journal.pone.0268120 (PMC9894398; doi:10.1371/journal.pone.0268120)
Supplement: Attachment — Submitted filename: response-to-reviewers-II.docx [file pone.0268120.s003.docx]

Barcelona 14/04/2022

Dear Dr. Shahid Farooq,

We hereby submit our revised manuscript ***Small-scale farming in drylands: new models for resilient practices of millet and sorghum cultivation*** for your consideration.

The revised manuscript with track changes has been compiled in LaTex using the latexdiff package. In the resulting PDF, removed text is in red and added/modified text in blue.

Please, find below a point-by-point response to the latest comments.

- Scientific name of maize is spelled incorrectly in introduction line 6. – **This has been corrected**
- The table legends must be above the tables, not below. - **This has been corrected**
- The figures must be incorporated within the text, like tables. – **Figures have been added (to add them to the LaTeX we had to use the .png and .pdf versions of the files. The files uploaded separately in the previous round are exactly the same just in .tiff version)**
- Agriculture in drylands heading should be deleted. This gives the impression that the paper is a review article. – **The heading has been deleted**
- In Table 1, the first letter of second word, i.e., agriculture must be written small caps in column 1 – **This has been changed**
- The n and f in Table 2 must be explained in footnotes - This has been added to the caption
- The scientific names of all species in the references section must be italic. – **This has been corrected (the change is not reflected in the manuscript with differences highlighted as the bibliography is a separate LaTeX file that is not checked when producing the diff.tex file)**

Please let us know if we need to change anything else.

Best wishes,

Carla Lancelotti

On behalf of all co-authors
